# Dual career policy at federal universities in Brazil: analysis of academic and sporting support

**DOI:** 10.3389/fspor.2024.1453749

**Published:** 2024-09-19

**Authors:** Junior Vagner Pereira da Silva

**Affiliations:** Centro de Desenvolvimento de Pesquisa em Políticas de Esporte e de Lazer da Rede Cedes no Mato Grosso do Sul, Faculdade de Educação, Curso de Educação Física, Faculdade de Medicina, Mestrado em Saúde e Desenvolvimento na Região Centro Oeste, Universidade Federal de Mato Grosso do Sul, Campo Grande, Brazil

**Keywords:** public politys, university sport, federal universities, dual career, holistic model, academic support, sporting support

## Abstract

Internationally, the implementation of holistic programs aimed at academic, financial and retirement preparation support for student-athletes who find themselves in dual careers, such as university students, has been widespread and intensified. However, little is known about the subject in the Brazilian national context, because although there are related studies, they are punctual and related to specific universities. In view of the above, this study aimed to investigate public policy aimed at student-athletes at Brazilian federal universities. This is an exploratory, qualitative, cross-sectional and documentary study, using the websites, internal regulations and public notices of 69 Brazilian federal universities for the year 2023, using the content analysis technique. The results indicate that 72.05% offer some kind of support to DC based on the holistic model, 66.17% of which is academic and 60.29% sports. Among the academic support, the flexibility of assessments (57.35%) and the differentiated treatment of absences (51.47%) were close. The sporting support is centered on partial payment of competition costs (60.29%) and athlete grants are restricted to 20.58% of universities. It can be concluded that Brazil's federal universities have policies that include support actions that integrate the holistic model (academic), with incipient coverage of scholarships (sports), but do not effectively follow the holistic model.

## 1 Introduction

It takes an average of five to ten years for an athlete to become part of the sporting elite (1), peaking between the ages of 20 and 30 (2). This age group has with a high percentage of university-educated athletes in South Africa (3), Belgium (4), Spain, Latvia, Italy, Portugal, Romania (5), the United States (6), Turkey (7), Spain (8) and Sweden (9). Therefore, people who find themselves in a dual career (DC) (10).

Dual career consists of combining a sporting career with studies or work (11). In this context, they accumulate the roles of student-athletes and their routines are permeated by numerous competing demands. In addition to the obligations inherent in a sporting career (physical, tactical and technical training, concentration, travel, friendly matches and competitions), they are also faced with the academic scenario, which has several other demands (fulfilling credits in subjects, studying to prepare and fix content, out-of-class work (12). This complexity is not linear, and it varies according to sporting level (amateur, semi-professional and professional) (13), which means that some dedicate 17.4 h a week (professionals) and others, 16.6 h (amateurs) (14). Macro issues (cultural and political) are also conditioning factors, which means the time dedicated to sport varies—Asia (27.6 h), Oceania (23.9 h), America (21.4 h), Europe (20.9 h) and Africa (14.7 h) (15). This is similar between countries, with a weekly dedication to sports of between 20 and 30 h in the United States (16), 11–20 h in Turkey (7), 6–9 h in public universities or over 10 h in private universities in Brazil (17). In addition, student-athletes are subjected to academic demands, which vary from less than 11 h/week (7) to 20 h/week (15).

This complex context, conditioned by various factors, has often overloaded student-athletes and resulted in incompatibilities, such as a lack of time to study, attend classes and reconcile exams and competitions (18), damaging their sporting careers with reduced training (15) and abandonment of their sporting careers (19). It has also had an impact on academic training, with absences from classes (7, 15), delays, failures, changes of shift (or university) and prolonged absenteeism from the course (7). In addition, the problems have gone beyond sporting and academic limits, because leisure time is limited (7, 15, 20), reconciliation with other daily experiences—studying, working, social relationships and leisure—is impaired (21). This is because the more student-athletes prepare and the more hours they dedicate to practicing and competing in sport, the less favorable the conditions for professional training become (22).

In view of this, in order to mitigate the side effects, the implementation of actions based on the holistic model, which consists of the reciprocal interaction between athletic, psychological, psychosocial and academic, and vocational development (23, 24) has been advocated for some decades, consisting of actions related to academic, sporting and transition to retirement aspects. These measures are essential for successful DC, given that DC is a multifactorial element, influenced by individual (talent and psychological characteristics), interpersonal (social support and culture), environmental (tools and services available) and political (national and international standards) aspects (25).

With regard to academic activities, the holistic model recommends making entry requirements more flexible, extending the school term, personalized study schedules, alternative access to course offerings, individual or small group classes. As for sports, the suggestions include scholarships, professional support, infrastructure for elite sports and elite sports development programs. As for the transition to retirement, the guidelines focus on scholarships, the introduction of new programs adapted for retiring athletes and lifestyle management services (26).

Faced with these demands and the possibilities of holistic interventions, initiatives aimed at a culture of DC have been implemented on the international stage. In the European Union, the Member States have made DC one of the strategic and political priorities of sport, including it in sports documents (27) and specific guidelines created for this purpose (28–30). In addition, transnational programs have been implemented, such as Program European Community Action Scheme for Mobility of University Students (ERASMUS + Sport) (31), Empathy (32); Dona, Starting11, Athletes2Business, Europeanh Athete as Student, More Than Gold (31), with adaptations to entry requirements, flexibility of schedules and exam dates in France, Hungary, Spain, Portugal (33).

These programs can serve as a basis for implementing DC-oriented policys in other international contexts, such as South America. However, they sometimes come up against the particular regulations of each country, as shown by Capranica et al. (34). In other words, polity is a conditioning factor for the policy model undertaken in each country.

This can be seen in Brazilian politics, where although in the past there were decrees granting academic support to student-athletes, there are no longer any. Since 1977, participation in sports competitions has been considered school attendance (35), but in 1993 it was revoked (36). Absences resulting from participation in national and international competitions between 1971 and 2019 could be excused by the Ministry of State for Education (37), but in 2019 the decree was revoked (38). Subsequently, these benefits were no longer included in any other sports or education law in the country (36, 39, 40).

On the other hand, financial support is provided by the Scholar-Athlete Program, which was implemented in 2004. Initially made up of five categories (basic, student, national, international and Olympic and Paralympic), student-athletes, in particular, can be included in the student category (41, 42).

Due to the lack of federal laws regulating academic support, such as flexible class attendance and the replacement of assessments missed due to participation in competitions, DC policy in Brazil is developed on an individual basis, with each institution's own initiatives. This is because, as regulated by the Brazilian Federal Constitution (43), universities enjoy didactic-scientific, administrative and financial and asset management autonomy. Therefore, as long as they do not violate the Federal Constitution, federal laws and/or decrees, universities can create their own policy, including those related to DC, academic support or sports.

However, in Brazil specifically, little is known about DC, because even though there are contributions on the subject, they are limited to the particular realities of specific universities, such as the University of Brasilia (44, 45) and the Federal University of Mato Grosso do Sul (12, 46, 47). An exception is the study by Quinaud et al. (17), which included a representative group of student-athletes taking part in the Brazilian University Games (JUBs), including those from public and private universities, but focusing only on identity issues.

Therefore, unlike what is evident in the international literature, to date there has been no research that has focused on a comprehensive analysis at the national level in Brazil, a gap that gave rise to the academic agenda in question. The data from this study could corroborate the debate on the subject in bodies responsible for educational and sports policy in the country, as well as broadening the international debate with an expanded scenario.

In light of the above, this research aimed to analyze policys focused on DC in Brazilian federal universities. Specifically, investigated it aimed the academic and sporting support directed at student-athletes in the context of Brazilian Federal Universities. It was hypothesized that the recommendations of the holistic model are present in an individualized and incipient way in the policys of Brazilian federal universities through academic e sporting support especific.

## 2 Materials and methods

### 2.1 Design

This is an exploratory study with a descriptive (48), cross-sectional (49) approach and qualitative-quantitative analysis (48).

### 2.2 Information sources

The research population was Brazil's federal universities. The sources of information were online documents related to the dual career sport policy. Of the 69 federal universities (50), 68 took part in the survey. Among them, 20 are located in the Northeast region, 19 in the Southeast region, 11 in the North region, 11 in the South region and 7 in the Center-West region.

### 2.3 Analysis technique

The analysis technique was documental, which consists of written records that provide information for understanding facts and relationships, making it possible to know the historical and social period of the actions and reconstruct the facts and their background. Written institutional records are those provided by government institutions (48). With regard to research into DC, document analysis (politys, regulations and plans) is an excellent methodological tool for discovering structural anchoring in national contexts. To provide a baseline and influence the phases of implementation projects that involve primary data collection, desk research includes the collection and content analysis of documents containing information on politys, regulations and plans, providing insights into problems and challenges (31).

In carrying out the research, the parameters indicated by Gil (48) were adopted, with organization in stages.

✓ Formulation of the problem: built up with the preparation of the research pre-project, based on the literature review, an action which, according to Deslandes (51), constitutes exploratory research.
✓ Drawing up the work plan: a preliminary and provisional stage in the process, consisting of drawing up the research project (48), drawing up the timetable (temporal organization of the bibliographical survey, literature review, data collection, analysis and interpretation of the results and preparation of the article for publication).
✓ Identification of Sources: classic documentary sources include public archives and official documents, trade associations, political parties, trade unions and scientific associations (48). Based on an exploratory review of studies already produced on the subject, the sources under investigation were identified: (a) Didactic regulations for undergraduate courses at Brazilian Federal Universities; (b) Sport and leisure politys at Brazilian Federal Universities; (c) Calls for proposals and results for scholarships and financial aid at Brazilian Federal Universities.
✓ Locating sources and obtaining material: based on the access to information law, consultations were carried out with Brazilian federal universities via the fala.br platform. Information was requested the existence of scholarships for training and participation of student-athletes in sports competitions; financial aid grants for participation of student-athletes in sports competitions; existence of academic regulations granting compensation for absences and/or replacement of exams to student-athletes on occasions of participation in sports competitions; existence of admission (entrance exam or similar) to the undergraduate program. In addition, it was requested that, if any of the concessions existed, the regulations and, in the case of scholarships and financial aid, the opening and results notices be made available.
✓ Data analysis and interpretation: the analytical procedures are different depending on the type of design, with content analysis being the most widely used technique for objectively, systematically and qualitatively describing transcribed communications (48). The following steps were followed in the content analysis: (a) definition of objectives; (b) creation of a reference framework; (c) selection of documents to be analyzed; (d) construction of a system of categories and indicators; (e) definition of analysis units; (f) definition of enumeration rules; (g) validity and reliability test; (h) data processing; (i) data interpretation.

Defining the objectives was one of the steps in the analysis process, which was developed in conjunction with the research problems. The frame of reference was established using the guiding assumptions of the holistic model of DC (24, 25), although, obtaining data related only to academic and sporting (26, 33). Another important step was the creation of categories and indicators. In constructing the system of categories and indicators, they were defined after reading the selected material. The categories were made up of key terms indicating the central meaning of the concept and the indicators the variations of this concept. The categories analyzed (variables considered) were: existence of CD policies at federal universities; characteristics and specificities of the academic support (type of academic support, type of standardization of academic support, type of absence compensation); characteristics and specificities of the financial support (target audience, type of regulation, application of financial aid, sources of funding), characteristics and specificities of the grants awarded (target audience, source of funding, scholarship amount, weekly training hours). Absolute frequency (number of cases) and relative frequency (percentage) analysis was applied to the data. Absolute frequency (number of cases) and relative frequency (percentage) analysis was applied to the data.

As this is a documentary study, following the guidelines, it was not necessary for the study to be submitted to and assessed by a Research Ethics Committee.

## 3 Results

**Figure 1:**
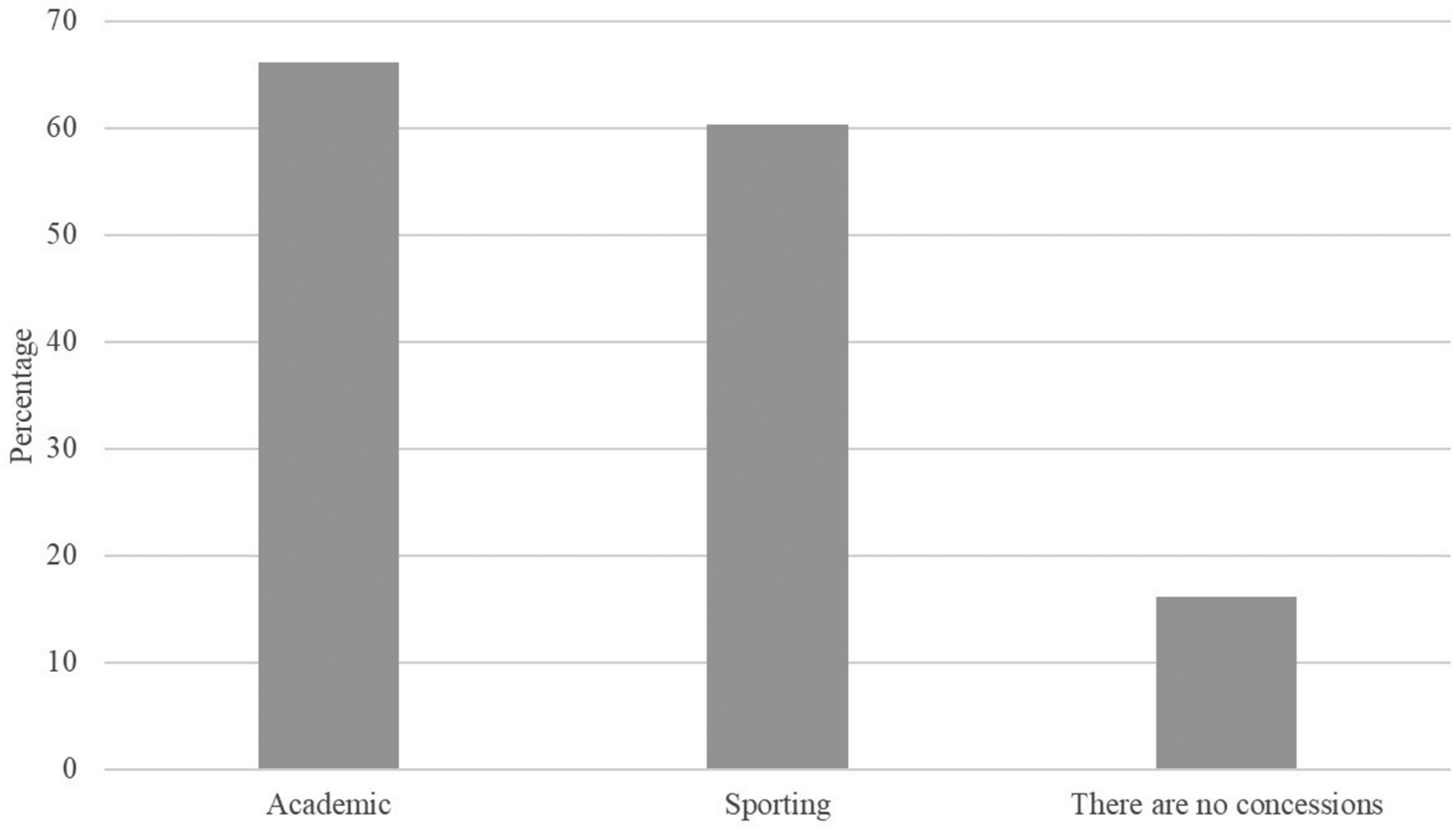
Percentage of occurrences by type of aid granted to student-athletes at Brazilian federal universities.

Of the 68 federal universities participating in the survey, 72.05% offer some kind of support to student-athletes, with a predominating two types—academic (differentiated assistance in relation to absences and evaluations) and sporting (payment of scholarships and financial aid for participation in competitions). However, academic concessions stand out, because they are present in 66.17% (Figure 1).

**Figure 2:**
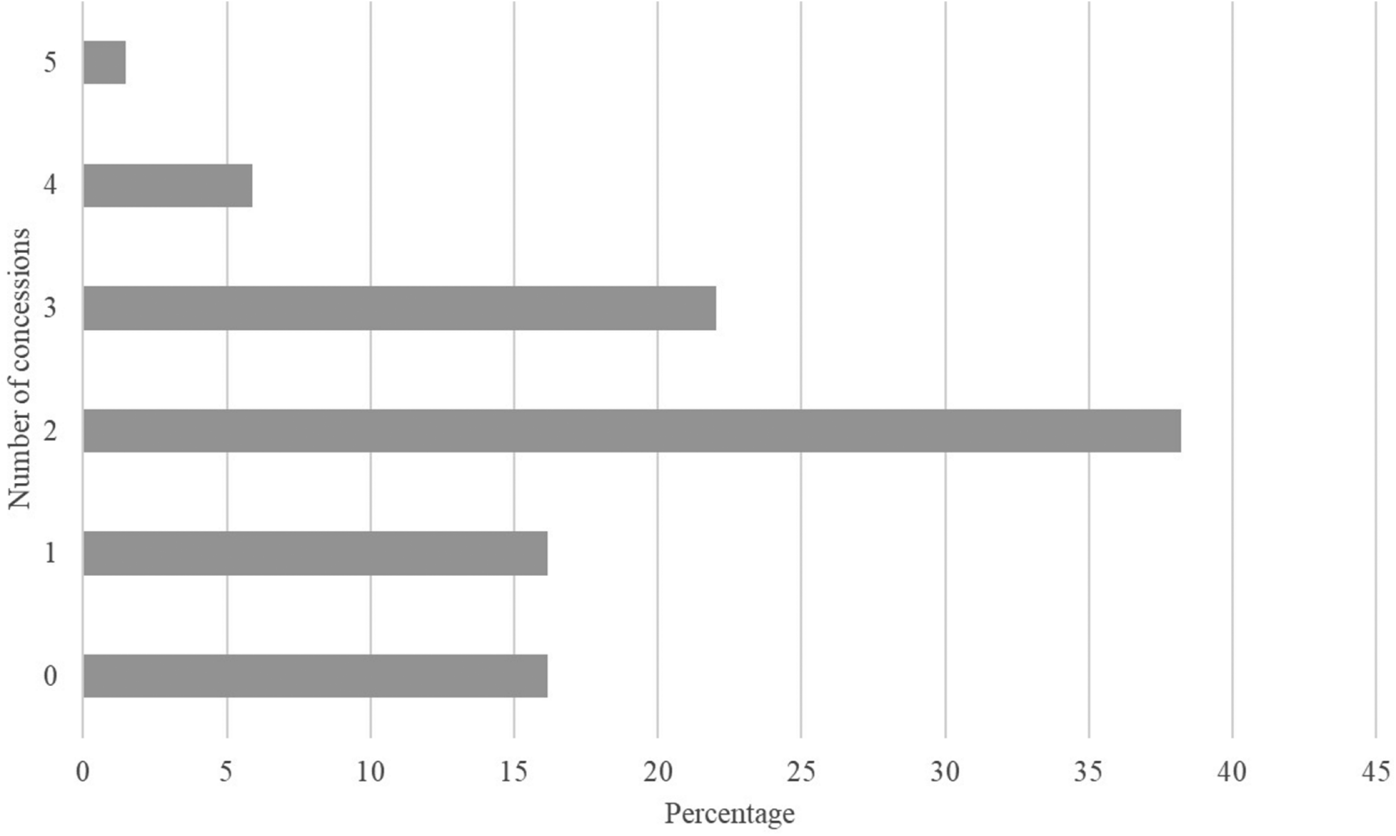
Percentage of occurrences by amount of aid granted to student-athletes at Brazilian federal universities.

Despite different types of measures created to mitigate existing barriers to dual careers (absence in classes, assessments on alternative dates, specific entrance exams for student-athletes, scholarships and financial support for participation in events), the granting of only two types of specific services (38.23%) was the most frequent (Figure 2).

### 3.1 Academic support

**Figure 3:**
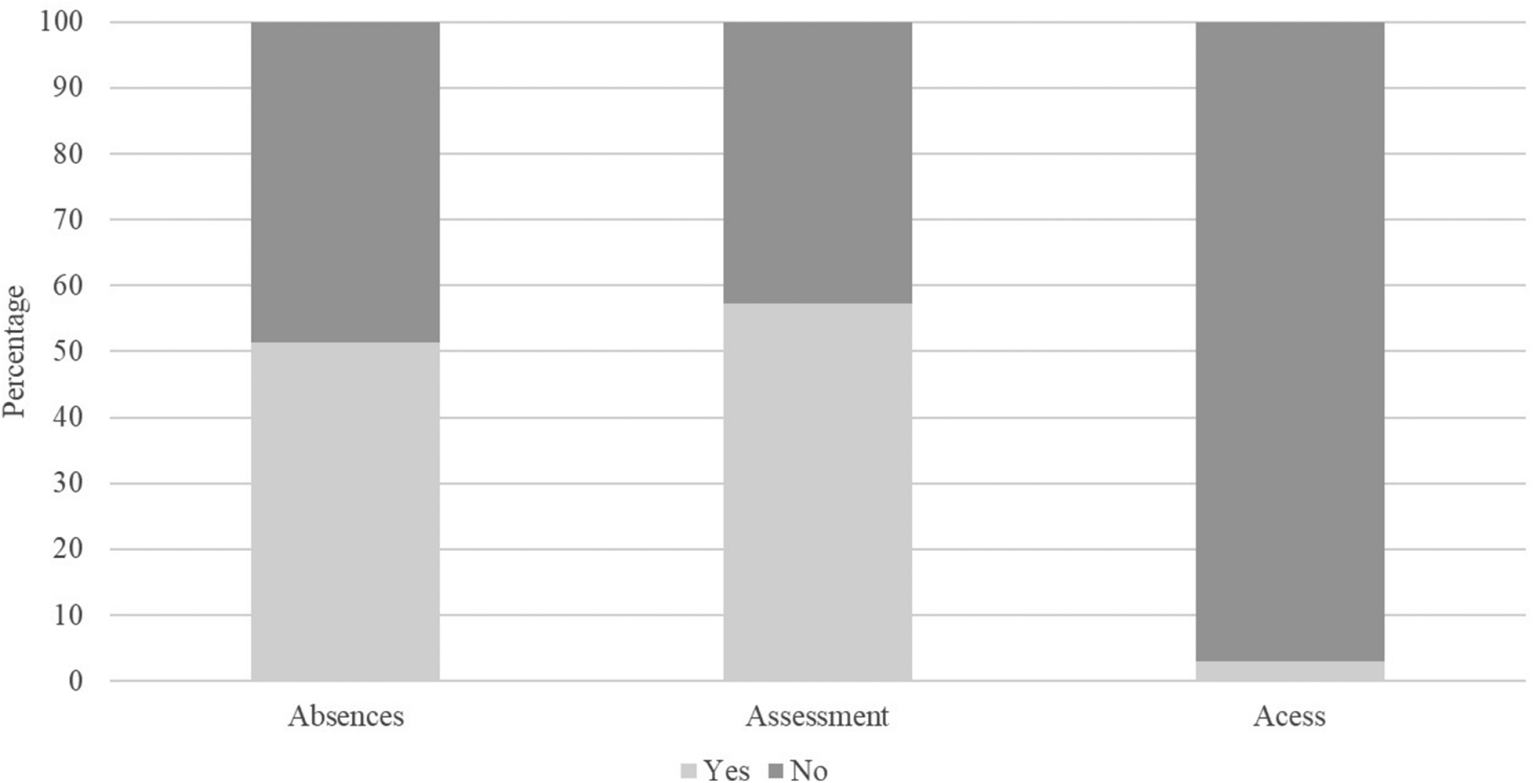
Percentage of occurrences by type of sports aid granted to student-athletes at Brazilian federal universities.

Academic support are present in 61.17% of the universities analyzed. Among them, the flexibility of assessments predominated (57.35%), with student-athletes being able to take them at another time when unable to due to sports competitions. Differential treatment in relation to absences showed similar percentages (51.47%) (Figure 3).

Regarding the types of benefits granted for absences from classes as a result of sports competitions, 35 universities that offer this service, with predominance of compensating absences through the universities’ own regulations (40.00%). However, there were also absenteeism allowances regulated by the universities (22.85%) and allowances conditional on the teacher's decision (20.00%). However, only 40.00% universities compensate for absences through a study plan, in which the student carries out academic activities home-based academic activities based on a study plan with activities prescribed by the lecturers.

In relation to assessment activities, 39 universities grant this benefit. Most of them (74.35%), have the right regulated by internal regulations, with a new assessment carried out (94.87%), in the second call format (62.86%) (Table 1).

**Table 1:** Types of academic support granted to student-athletes at Brazilian federal universities.

Description	Absences		Flexibilization evaluations	
	*N*	%	*N*	%
Type of academic support				
Allowance regulated by universities	8	22.85	–	–
Compensation regulated by universities	14	40.00	–	–
Justification regulated by the IFES	3	8.57	–	–
Allowance conditional on teacher's decision	7	20.00	–	–
Allowance based on extinct laws	2	5.71	–	–
Evaluation standardized by IFES	–	–	29	74.36
Evaluation at the teacher's discretion	–	–	4	11.43
Evaluation based on extinct laws	–	–	4	11.43
Verification work/exercises	–	–	2	5.71
Not specified	1	2.94	–	–
Type of standardization of academic support				
Specific leave	1	2.86	1	2.56
Leave of absence	8	22.86	–	–
Justification	6	17.14	1	2.86
Reduced schedule	1	2.86	–	–
Home regime	9	25.71	7	20.00
Accompanied activities	1	2.86	1	2.86
Exceptional treatment	1	2.86	–	–
Specific exercise regime	1	2.86	1	2.86
Content and attendance replacement scheme	1	2.86	–	–
Special arrangements	1	2.86	–	–
Academic leave	2	5.71	2	5.71
Compensation	1	2.86	–	–
Second call	–	–	22	62.86
Exceptional learning assessment	–	–	1	2.86
Special learning regime	–	–	1	2.86
Special exam	–	–	1	2.86
Replacement of work	–	–	1	2.86
Not specified	1	5.72	2	5.71
Type of absence compensation				
Evaluation	–	–	37	94.87
None	15	42.86	–	–
Study plan	14	40.00	–	–
Academic activity	1	2.86	–	–
Work/checking exercise	1	2.86	2	5.40
Not specified	4	11.42	–	–

### 3.2 Sporting support

**Figure 4:**
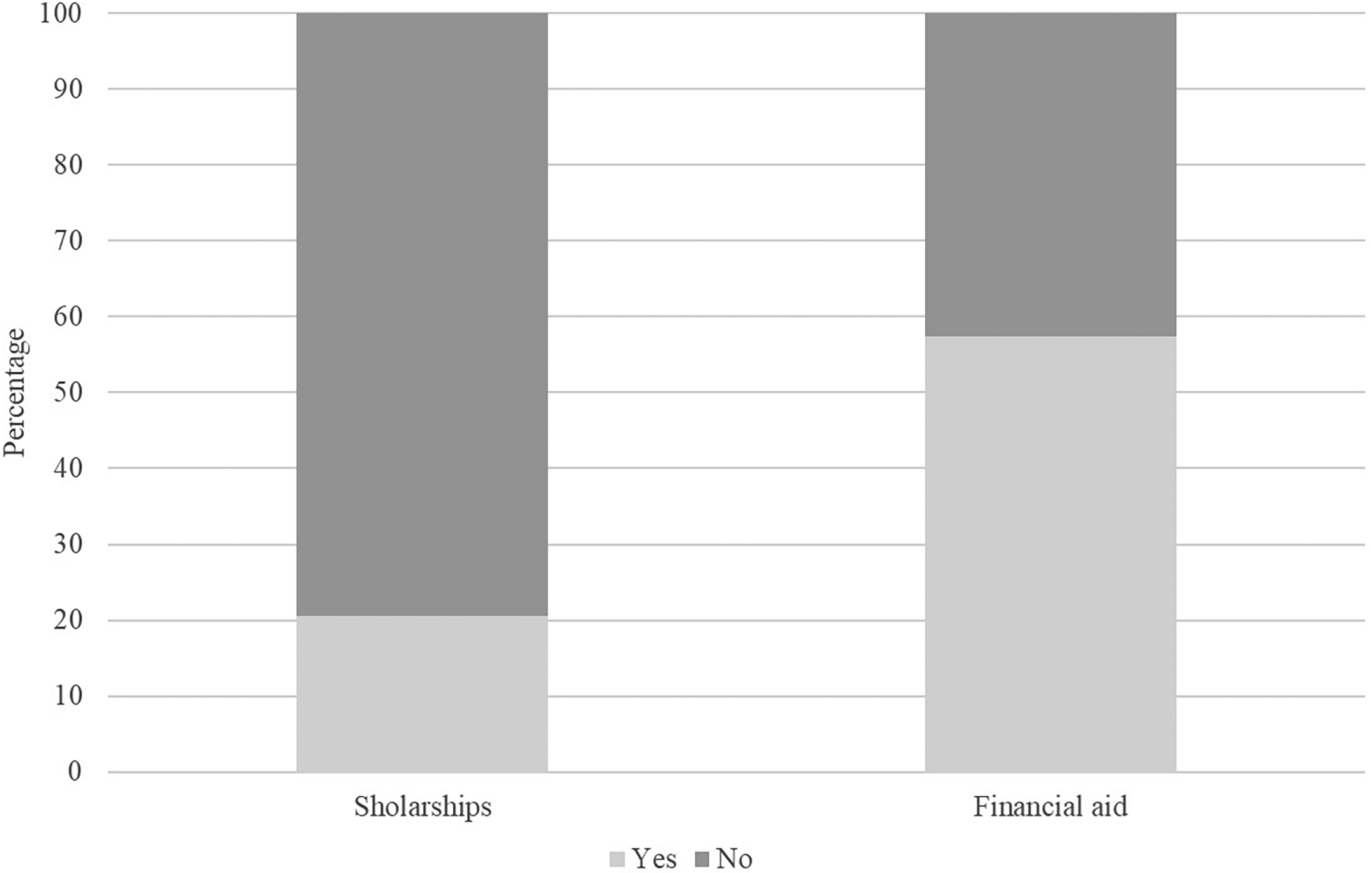
Percentage of occurrences by type of sports aid granted to student-athletes at Brazilian federal universities.

Among the 41 Federal Universities that have some action aimed at sporting support, there is a predominance of financing participation in sports competitions (57.35%) compared to the payment of scholarships (20.58%) (Figure 4).

Of the 39 federal universities that provide financial aid, the target audience is predominantly undergraduate students (38.46%), using funds from the National Student Assistance Plan (41.02%). In most Brazilian federal universities, these benefits are the subject of general public notices, which encourage participation in academic-scientific, cultural, student representation and sporting events. Among the units of expenditure, payments for transportation (61.53%), registration in competitions (56.41%) and accommodation (53.84%) predominate (Table 2).

**Table 2:** Characteristics and specificities of the financial support given to student-athletes at Brazilian federal universities.

Description	Financial support	
	*N*	%
Target audience^a^		
Undergraduate students, primarily in socio-economic vulnerability	15	38.46
Postgraduate students	6	15.38
Undergraduate students	12	30.76
Not specified	12	30.76
Servants	1	2.56
Scholar-athletes	1	2.56
Postgraduate students, with priority given to socio-economically vulnerable students	1	2.56
Type of regulation		
General (teaching, research, extension and sport)	19	48.71
Specific to sports	11	28.20
At the request of the higher administration	9	23.07
Application of financial aid^a^		
Accommodation	21	53.84
Food	18	46.15
Transportation	24	61.53
Registration in competitions	22	56.41
Uniforms/equipment	4	10.25
Medical examination	1	2.56
Health insurance	1	2.56
Allowance	1	2.56
Sources of funding^a^		
National Student Assistance Plan	16	41.02
Others headings	2	5.12
Others sources	1	2.56
Not specified	18	46.15
Institutional representation fund	1	2.56
Own sources	3	7.69
Union lines	1	2.56
Parliamentary amendments	1	2.56

^a^ Variables identified in the documentary analysis of more than one alternative.

As with financial aid, the main target group for grants were undergraduate students (71.42%), especially those in vulnerable situations (42.85%) and primarily those in socio-economic vulnerability (28.57%). This is related to the National Student Assistance Plan being the main source of funding (64.28%) for the scholarships. The value of the 50% grant was 62.57€, with the most frequent schedule being 12 h (28.57%) (Table 3).

**Table 3:** Characteristics and specificities of the grants awarded to student-athletes at Brazilian federal universities.

Description	Financial support	
	*N*	%
Target audience^a^		
Undergraduate students	10	71.43
Postgraduate students	1	7.14
Undergraduate students, with priority given to socio-economically vulnerable students	4	28.57
Socioeconomically vulnerable undergraduate students	6	42.86
Distance learning undergraduate students	1	7.14
Stricto *sensu* postgraduate students	2	14.29
Source of funding		
National Student Assistance Plan	9	64.29
Not specified	3	21.43
Parliamentary amendments	1	7.14
Own resources	1	7.14
Scholarship amount^a^		
31.96€	1	7.14
49.18€	1	7.14
65.57€	7	50.00
86.06€	2	14.29
114.75€	2	14.29
Not informed	1	7.14
Weekly training hours		
6h	1	7.14
8h	3	21.43
12h	4	28.57
20h	2	14.29
Not informed	4	28.57

^a^ Variables identified in the documentary analysis of more than one alternative.

## 4 Discussion

This research aimed to analyze policys focused on DC in Brazilian federal universities. Specifically, it aimed the academic and sporting concessions directed at student-athletes in the context of Brazilian Federal Universities. In order to better analyze the data obtained, the discussion is presented in topics.

### 4.1 Academic support at Brazilian federal universities

Almost three quarters of Brazilian federal universities provide some kind of support to student athletes. These results are positive and exceed those seen in Colombia, which has 67.40% of the universities investigated, including public universities (67.00%), which together with institutions with a specific degree in Physical Culture, Sport and Recreation or Sports Sciences (70.00%), have the best results (52). On the other hand, data from Spain is more favorable in relation to Brazilians and Colombians, since of the 49 universities investigated, 79.60% had official support programs for student athletes (53).

Since 1990, Spain has had a regulatory framework focused on dual careers (1990 Sports Law, Royal Decree 1467 of 1997, Order of April 14, 1998, and Royal Decree 971 of 2007), making it a country that uses the state-centered model, with federal regulations and university obligations towards student-athletes defined in regulations (26). In the last 17 years, dual career has been included among the priorities of European sports strategies and politys (28–30), and has been accelerated by the Treaty on European Union since 2009 (26). In 2012, the Member States drew up the European Guidelines for dual university careers and, from the transnational creation of the guidelines, were directed to create national politys and guidelines, which may explain the normative structure and Spanish politys.

Therefore, unlike what happens in countries that have specific regulations centered on the state, such as France, Hungary, Luxembourg, Spain, Poland and Portugal (33), in Brazil, the laisser-faire model prevails (46), in which little or no formal structure is directed at DC (26). This situation stems from the fact that there are no federal laws or decrees in Brazil that deal directly with PD, whether in the field of education or sport.

The only mention of the subject occurs indirectly and in authoritative terms in the Pelé Law and the General Sports Law. Article 85 and article 206, respectively, state that

The education systems of the Union, the States, the Federal District and the Municipalities, as well as the higher education institutions, will define specific rules for verifying the performance and controlling the attendance [sic] of students who are part of national sports representation, in order to harmonize sports activity with the interests related to school achievement and promotion” (39, 40, s.p.).

In this context, in the absence of federal laws regulating DC, support to student-athletes depend on the creation of internal and specific rules by universities, including federal universities, which are entities of the executive branch that make up the state, but which, according to article 207 of the Brazilian Federal Constitution (43), enjoy didactic-scientific, administrative, and financial and asset management autonomy. As Durhan (54) points out, universities have the prerogative to regulate themselves by means of their own rules in order to fulfill the social purposes for which they were set up, with the Regulations and Statutes being the way in which they establish their own rules. In the case of federal universities, which are fully funded by the federal government, the relationship with government bodies is closer. However, even so, they are not a state body like the others, since they are sui-generis institutions.

In this sense, the presence of academic and/or sporting activities highlighted in this study stems from the political will and importance given to sport by the managers of each institution and, in particular, to performance sport practiced by DC students. The observance of political will towards student-athletes at the universities investigated is a positive and favorable factor in the Brazilian context, given that at an international level, according to Abelkalns et al. (5), university rectors and directors have been shown to be the least supportive of student-athletes.

In this sense, public politys promoted by the Executive (public administration), such as the Brazilian Federal Universities, through concrete actions such as the academic ones shown in this study, are materialized in political programs/government plans in which they define the contents (policy), which are conditioned by the normative dimension of the institutional structure of the political-administrative system (polity) and both, inserted in a broad and complex process of disputes between the different actors involved in the selection of objectives, contents and distribution of powers (politics).

#### 4.1.1 Flexibility in daily academic activities

Academic benefits are those that do not directly require the investment of financial resources. All that is needed is the political will of managers to propose and mobilize the internal support of representatives of the central administration, the sector and students so that the standardization of benefits at the institutional level takes place and the procedures to be adopted are outlined and implemented via resolutions. This could explainwhich may explain their predominance among Brazilian federal universities.

On the international stage, flexibility in daily academic activities allows student-athletes to fulfill academic activities on individual study and exam schedules in Hungary. In addition, they are provided with textbooks, manuals, special literature and other auxiliary educational materials that allow them to study during periods of training and competition (26).

The flexibility of academic issues dates back more than 21 years in Europe, as in 2003 initiatives were registered in Belgium, Germany, Denmark, Greece, Spain, France, Ireland, the Netherlands, Finland, England, Northern Ireland, Scotland and Wales (26). This condition is similar to the attention given to academic issues in private and public universities in Spain, since of the 49 participants in the research, 79.60% had official support programs and, among them, 69.60% had academic support, such as personalized tutoring and flexibility in teaching schedules and 78.30% allowed the change of assessment dates (53). The Catholic University of Murcia, Spain, promotes monitoring of academic and sporting performance, personal tutoring for information and ongoing advice on topics of interest (academic, personal and professional), coordination of sporting commitments and respective academic obligations, coordination of academic, administrative and economic procedures, access to the student tutor and access to the virtual system (55). In South America, Colombia promotes absenteeism (76.70%), flexible deadlines for assessment activities (62.80%) and exams (58.10%) (52), a condition also evidenced at the University of Santo Tomás (56).

Academic flexibilization in Brazil was present in federal regulations for more than 48 years (1971–2019), during which time Decree No. 69.053 of August 11, 1971 was in force, giving the Minister of State for Education and Culture the prerogative, by means of an ordinance, to validate the participation of students in national and international sports competitions as academic attendance (37). In this sense, it followed the general sports regulations (1977–1993), which in article 144, recognized participation in official student sports competitions as school attendance, limiting them to 180 days (international competitions), 90 days (national competitions), 60 days (state competitions) (35).

Despite the significant percentage of federal universities that have their own mechanisms for granting benefits to student-athletes, over the last 31 years there have been regulatory setbacks in university sports policy, since federal laws and decrees that ensure such rights have been repealed. Therefore, the federal support regulated by laws and decrees must be rethought and reincorporated back into Brazilian legislation, with parliamentary amendments to the General Sports Law enacted in 2023 being one of the possible avenues to be undertaken by the Sports Commission of the Chamber of Deputies, the body responsible for processing changes to sports-related laws at the federal level.

Although it is not a condition for universities to implement their own politys via internal rules, the creation of regulatory rules at the federal level and with the duty to comply by universities, will enable the benefits of programs based on the holistic model to be present not only in part, but in all Brazilian federal universities. This situation could increase the possibilities for athletes to enter higher education and enjoy greater and better conditions to progress in academic training and sports performance.

This is a high and present demand on the national (45) and international scene, given that a high percentage of Olympic athletes (Barcelona/1992, Atlanta/1996 and Sydney/2000 Olympic Games) experienced the dual career process when they were at a level of sporting excellence. In addition, the percentage of athletes with higher education at the time of retirement from sport was higher among those who went through dual careers (53.30%) compared to those who dedicated themselves exclusively to sport (14.50%) or work (27.30%) (8).

#### 4.1.2 Absence allowances and Brazilian regulations

Despite the prerogatives of university autonomy, it is clear that, among the Brazilian federal universities that grant leave of absence, five do so in the wrong way and contrary to federal regulations, as they are based on revoked decrees—n° 54.215/64 (57), n° 69.053/71 (37), n° 69.450/71 (58), n° 80.228/1977 (35)—when, in theory, they should be supported by current laws n° 9.615/98 (39) e n° 14.597/2023 (40). In addition, 15 universities grant the absence allowance without any kind of replacement or compensation, which is also illegal, since current legislation requires 200 days of effective academic work to be completed, and students must attend at least 75.00% of the course load (59, 60).

In addition to being illegal, the allowance of absences based on personal assessments, such as those in which there are no regulations and the teachers decide, as evidenced in this research at seven universities, is an inadequate and ineffective factor for DC. In this regard, Álvarez and Lópes (18) warn that when issues are left to the individual, with each athlete having to deal with the teachers, students often don't find the necessary support in requests for changes to assessment dates. The lack of sensitivity on the part of teachers (77.80%) at La Laguna University in Spain was among the main obstacles to reconciling academic and sporting life (61). In Kosovo, even though it is the main mechanism for academic achievement, support from teachers is infrequent (20).

Considering that Brazilian educational legislation prohibits the excusing of absences, except in cases that have legal authorization; that current sports legislation establishes that universities will create specific rules for harmonizing academic and sports training; the regulations with the inclusion of absences in a compensation process through academic activities at 14 universities proves to be an appropriate path to be followed by the others. Such allows students to recover lost content through home studies, favoring the maintenance of student-athletes in DC. Still allows as providing them with quality training, since promoting alternative learning conditions for the content worked on during the period of absence is essential.

Regarding qualitative care for student-athletes, it is important to note that internationally, such as in Hungary, textbooks, manuals, special literature and other auxiliary educational materials are adopted and allow study during periods of training and competitions (26). In the Brazilian Federal Universities can incorporate them into future programs, in addition to the study plan.

#### 4.1.3 Forms of admission for student athletes

Since the forms of admission are a decision established by the universities themselves because they have didactic-scientific, administrative and financial and asset management autonomy (43) and are responsible for establishing the criteria and rules for selecting and admitting undergraduate students (59), the creation of specific entrance exams for student athletes at the Federal University of Mato Grosso do Sul (UFMS) and the Federal University of Santa Maria (UFSM) in 2023/2024 demonstrates innovation in the DC scenario in Brazil. On the other hand, it suggests a late policy in relation to the international context, since 21 years ago (2003) student athletes from Germany, France, Finland and the United Kingdom (26), already had this benefit.

The UFMS entrance exam covers all types of student-athletes, whether at university representation, amateur or elite level (Olympic Games, Paralympic Games or Brazilian University Games). At the Federal University of Santa Maria, only athletes in training, between the ages of 16 and 23, with the potential to be a sporting talent in futsal, athletics, handball and volleyball, and former high-performance athletes who have had international prominence in sports recognized by the Brazilian Olympic Committee (62).

In addition to the model adopted by the two Brazilian universities (specific entrance exams), there are other specific mechanisms on the international scene for student-athletes to gain access to universities. In Spain, high-performance athletes who meet at least one of the requirements are allocated 3.00% of the places; in Hungary, Olympic medalists have the benefit of entering any university without having to take entrance exams; in Poland, athletes with exceptional sporting results, when referred by their federations to the Ministry of Education and Sport, do not have to take entrance exams (26, 33).

The academic initiatives (absences, flexibilization of assessments, specific entrance exams) seen in Brazilian federal universities for student-athletes are relevant national measures that can make important contributions to harmonizing academic training and sports performance, given the incompatibility of schedules between the two activities (5, 61) have a negative impact on academic life—lack of time to study (61, 63), delays, failures, changes of shift or academic abandonment (64)—and on sporting life—career abandonment (19).

### 4.2 Sporting support

Financial aid consists of sporadic concessions, which occur according to specific demands (actions, projects, programs or competitions), and can be promoted via general public notices (teaching, research, extension, sport, student representation), specific ones, such as sports or by demand of interest from the higher administration. Scholarships, on the other hand, are amounts paid monthly, with a defined value and period, and are not to be confused with employment, but rather a donation due to student participation in projects/programs.

The percentage of Brazilian federal universities receiving some kind of financial support was lower than that found in Colombia, where 74.40% of the institutions taking part in the study reported receiving it (52). However, they are positive compared to a study carried out in Spanish universities, of which only one of the 10 investigated had this device (65).

The provision of financial aid for participation in events such as sports competitions, as evidenced in this study, is essential for students, including those in DC, to have part of their transportation, registration, accommodation and food costs covered. In a study carried out by Abelkalns et al. (5) in Europe, financial support for participation in international competitions was identified by 48.00% as the fourth greatest need of student athletes. According to Albuquerque, Vasconcelos and Silva (46), financial aid can increase motivation to stay in DC, as well as encourage participation in a greater number of competitions, given that the cost of traveling to the city, state and international level to compete is high.

On the other hand, the granting of scholarships to student-athletes was not very present in Brazilian research, reaching only 20.58%. These results are lower than those observed in Spanish universities, which also do not have a significant percentage, limited to 34.80% (53). Low involvement in sholarships funding is common, and is also present in other locations, such as in Catalan institutions, where out of ten universities investigated, only one promoted this benefit (65) and at the University of Turin, only 1.68% of student-athletes are covered (66).

Although students at federal universities in Brazil have free access, the payment of scholarships is of great importance and can contribute to maintaining part of the expenses inherent in sporting and academic life. In Italy, Portugal, Latvia, Romania and Spain, financial support was considered fundamental, especially by those involved in sports with no income, which may be related to the fact that it gives them access to advantages they wouldn't otherwise have (34). Because they dedicate themselves to academic and sporting training concurrently, student-athletes are unable to incorporate another demand (work), because for many, even if there is pecuniary remuneration, sport is work itself (12).

The monthly income from dedication to academic and sporting training can contribute to the purchase of sports equipment, food, supplements, gym fees, medical treatment, among other things. Paz et al. (67) found that funds from scholarships were used to buy supplements, gym equipment and clothing and to pay for a nutritionist. In addition to sports expenses (tuition, sports materials, gyms), Lagos Cortes (52) showed that scholarships also help with academic costs (books, school materials) and personal costs (food, housing, transportation).

Therefore, the granting of this benefit is of great importance, as its loss would lead to sports dropout (13, 68) or academic dropout (18, 64). Parents of student-athletes from European Union Member States (France, Ireland, Italy, Portugal, Slovenia) point out that the maintenance of expenses by the family itself (academic and/or sports fees and expenses related to sports equipment, participation in competitions, transportation, accommodation, equipment classes, specialized training, academic support and extra academic classes) is a factor that makes it difficult to continue in DC (25). On the other hand, academic and sports scholarships make it easier to stay (69).

In addition to the low percentage of institutions offering scholarships to student-athletes at Brazilian federal universities, another limitation was the lack of a specific program to pay for them, since the main source used comes from the student assistance polic. The program was created in 2010, with the aim of increasing the conditions for young people to remain in federal public higher education, with universities being able, based on the creation of internal mechanisms of their own regulation, to apply the resources derived from it in actions of student housing, food, transport, health care, digital inclusion, culture, sport, daycare and pedagogical support (70). As this is a student assistance program and not specifically a DC policy promotion program, not everyone can benefit from it, because in addition to proving sports performance skills and physical athletic aptitudes, priority for assistance must be given to students from public high schools who have a per capita income of up to one and a half minimum wages, as is evident from the main target audience.

Given the limited conditions for funding scholarships via the National Student Assistance Plan, another alternative is the athlete scholarship from the federal program created in 2004 (71). Among the different categories, student-athletes can be included in the “student athlete” category, which is aimed at students aged 14–20, who are awarded up to third place in individual sports or are elected among the six (6) best athletes in each team sport in national student events organized in the previous year, directly or indirectly, by the Brazilian Olympic Confederation, the Brazilian Paralympic Confederation, the Brazilian School Sports Confederation (CBDE) and the Brazilian University Sports Confederation (CBDU), recognized by the Ministry of Sport, provided they continue to train and take part in national competitions (40).

Despite appearing as an alternative, the “student athlete” category has a limit of 21 years. This makes it difficult hardly covers student-athletes at university level in Brazil, as the majority of participants the Brazilian University Games (JUBs) in 2020 (54.00%) and 2022 (54.00%), were over the age of 21 (72, 73).

With regard to the amount allocated to scholarships, 65.57€ prevailed, which is higher than the amount financed by the Ministry of Sport in the “student athlete” category of the federal Bolsa-Atleta program, which since 2011 has financed 12 installments of 60.65€ (74). Although the efforts made by the managers of Brazil's federal universities to implement scholarships for student athletes are important and relevant, even operating with amounts higher than those practiced by the Ministry of Sport, when compared to the costs inherent to DC, the amount is derisory, given the various expenses arising from the accumulation of academic and sporting activities.

In addition to being derisory, the payment of scholarships in the context of Brazilian federal universities is discontinuous. Because the financial resources come from transfers from the Ministry of Education, which begin annually in April (46), the maximum paid has been 9 months, which is a problem, since in order to achieve the desired sporting performance, student-athletes are subjected to training on average 5.7 days/week in individual sports and 4.6 days/week in team sports (14), for 6–9 h at public universities (17). In this sense, increasing the value of the scholarship to 114.75€, as some of the federal universities analyzed already operate, would be a feasible alternative, since the value of the scientific initiation scholarship operated by the National Council for Scientific and Technological Development is a parameter for other scholarships in the context of Brazilian higher education (46) and, since February 2023, the entity has readjusted the scientific initiation scholarship to 114.75€ (75).

The requirement to dedicate at least 12 h to sports training in most universities indicates that this is lower than that observed in amateur, semi-professional and professional student-athletes (14). However, they are higher than those observed in a Brazilian study of public and private universities (19), which may be related to changes in politys aimed at DC at federal universities.

The hypothesis initially raised that the support academic and sporting of the holistic model are present in an individualized and incipient way in Brazilian federal universities through specific support, was partially refuted. This is because although in the Brazil there is no national polity aimed at DC (laisser-faire model), the support academic is present in most universities, with only the granting of athlete scholarships being incipient.

Despite the advances in scientific production promoted by the research in question, highlighting the DC policy in all Brazilian federal universities, as it was not the object of the investigation, the profile of student-athletes was not known, which appears as a limitation. Another limitation was the restriction of the analysis contained in documents, which did not make it possible to delve deeper into the issues related to the student-athletes’ perception of the policy undertaken at Brazilian federal universities, as well as other types of support that integrate the holistic model in the political aspect. Another limitations were difficulties in accessing the documents of each university, challenges in comparing them, and the dynamic nature of the data, which may have changed between the time it was gathered (2023–2024) and now (2024–2025).

Therefore, it is necessary that future research be developed directly with student-athletes in order to outline their demographic and sporting profile, enabling a greater understanding of the proximity or distance between the DC policies of Brazilian federal universities and those implemented at an international level, condition that is necessary, as age (junior vs. adult), structural/organizational (culture and university export policy in each country) and athletic proficiency (regional vs. national; national vs. international) transitions may differ around the world. Likewise, it is also appropriate for future investigations to produce data regarding other factors that make up the holistic dual career model, in other types of support in polity, as well as other aspects (individual, interpersonal and environmental) are analyzed.

### 4.3 Practical implications

This research is the first to analyze data from all Brazilian federal universities. Since evaluations of public policies are a mechanism for guiding public managers in obtaining results and optimizing public resources, improving decision-making in the allocation of resources, the data obtained can corroborate the design of policies aimed at DC. At a micro level, universities that have no support can mirror those that already have a certain structure. Those that already have actions in place can expand the existing types of support. At a macro level, the findings of this research could encourage discussions in political arenas, such as the legislature, in order to create laws that regulate the flexibilization of teaching activities when student athletes take part in official competitions.

## Data Availability

The raw data supporting the conclusions of this article will be made available by the authors, without undue reservation.
